# Detection of circulating tumor DNA from non‐small cell lung cancer brain metastasis in cerebrospinal fluid samples

**DOI:** 10.1111/1759-7714.13300

**Published:** 2020-01-13

**Authors:** Chunhua Ma, Xueling Yang, Wenge Xing, Haipeng Yu, Tongguo Si, Zhi Guo

**Affiliations:** ^1^ Department of Interventional Therapy Tianjin Medical University Cancer Institute and Hospital, National Clinical Research Center for Cancer, Key Laboratory of Cancer Prevention and Therapy, Tianjin's Clinical Research Center for Cancer Tianjin China

**Keywords:** Brain, cerebrospinal fluid, circulating tumor DNA, metastasis, next‐generation sequencing

## Abstract

**Background:**

Evaluating the molecular characteristics of brain metastases is limited by difficult access and by the blood–brain barrier, which prevents circulating tumor DNA (ctDNA) from entering the blood. In this study, we aimed to compare the sequencing results from cerebrospinal fluid (CSF) ctDNA versus plasma ctDNA, plasma circulating tumor cells (CTCs), and brain tissue specimens from patients with brain metastasis from non‐small cell lung cancer (NSCLC).

**Methods:**

This was a prospective study of 21 consecutive patients with NSCLC and brain metastasis diagnosed between April 2018 and January 2019. Samples of CSF and peripheral blood were obtained from all 21 patients. Brain tissues were obtained from five patients after surgical resection. Next‐generation sequencing was performed using the Ion system. Single nucleotide variants (SNVs) and small insertions or deletions (indels) were searched.

**Results:**

Mutations were detected in the CSF ctDNA of 20 (95.2%) patients. The detection rate of epidermal growth factor receptor (*EGFR*) mutations in CSF ctDNA was 57.1% (12/21) whereas this rate was only 23.8% (5/21) in peripheral blood ctDNA and in CTCs. *EGFR* mutations were found in the CSF of 9 of 11 (81.8%) patients with leptomeningeal metastases, as compared with three of 10 (30%) patients with brain parenchymal metastases. Mutations were also detected in *KIT*, *PIK3CA*, *TP53*, *SMAD4*, *ATM*, *SMARCB1*, *PTEN*, *FLT3*, *GNAS*, *STK11*, *MET*, *CTNNB1*, *APC*, *FBXW7*, *ERBB4*, and *KDR* (all >10%). The status of *EGFR* and *TP53* mutations was consistent between CSF ctDNA and brain lesion tissue in all five patients.

**Conclusion:**

Sequencing of CSF ctDNA revealed specific mutation patterns in driver genes among patients with NSCLC and brain metastasis.

**Key points:**

In some small‐sample studies, the importance of cerebrospinal fluid in guiding the treatment of cancerous brain lesions has been verified in that it may reflect genomic mutations of brain tumors relatively accurately.

Cerebrospinal fluid is a new form of liquid biopsy that can be helpful in improving the management of patients with brain metastasis from non‐small cell lung cancer by detecting genetic abnormalities specific to brain metastases.

## Introduction

Brain metastasis is a common neurological complication of systemic cancer in which metastases from the primary extracranial tumor invade the brain, usually indicating end‐stage disease and short life expectancy.^1^, ^2^ The reported annual incidence of brain metastases is 8.3–14.3 per 100 000 people.^3^ Brain metastases are found in 8%–20% of patients with cancer, but the frequency is even higher on autopsy.^1^, ^2^, ^4^ In addition, brain metastasis is 10 times more common than primary brain tumor.^1^, ^2^, ^4^ Primary cancers that are likely to metastasize to the brain include non‐small cell lung cancer (NSCLC) (24%–44%), small cell lung cancer (6%–15%), breast cancer (13%–30%), melanoma (6%–11%), gastrointestinal cancer (6%–9%), and colorectal cancer (4%–8%).^3^

Examining the molecular characteristics of brain metastases may assist in guiding therapy as the tumor clone that metastasizes to the brain may be different from the primary tumor.^5^, ^6^ In NSCLC, brain metastases respond poorly to conventional treatments.^7^ In addition, occult primary cancers are responsible for 2%–14% of cases of brain metastasis.^3^ However, obtaining tissue specimens from brain lesions is nearly always impossible. Furthermore, the blood‐brain barrier prevents circulating tumor DNA (ctDNA) from brain lesions to pass into the blood circulation, and ctDNA found in the peripheral blood will mostly reflect the primary tumor and metastases at other extracranial sites.^8^ Therefore, in patients with brain metastases, cerebrospinal fluid (CSF) ctDNA could better represent the molecular status of intracranial lesions, to help guide clinical targeted treatment.

A study by Pan *et al*.^9^ demonstrated that sequencing of CSF ctDNA in patients with brainstem glioma could characterize molecular alterations of the tumor more comprehensively than tissue biopsy from a single site, and the results were superior to those of blood ctDNA. The genomic and molecular characteristics of brain lesions change with treatment and treatment line progression. Tumor load can be evaluated using imaging, such as magnetic resonance imaging (MRI), but CSF liquid biopsy could facilitate dynamic monitoring of the therapeutic effect on brain lesions, indicating the prognosis. Detection of CSF ctDNA is convenient, comprehensive, and less invasive for the diagnosis of brain lesions and may offer an alternative method to stereotactic biopsy.^9^

The importance of CSF in guiding the treatment of cancerous brain lesions has been verified in some small‐sample studies showing that it could relatively accurately represent the genomic mutation of brain tumors.^10^, ^11^, ^12^, ^13^, ^14^ Nevertheless, data on brain metastases are still very limited. Therefore, the objective of the present study was to compare the results from CSF ctDNA with plasma ctDNA, plasma circulating tumor cells (CTCs), and brain tissue specimens in patients with brain metastasis from lung cancer.

## Methods

### Study design and patients

This was a prospective study of 21 patients with NSCLC and brain metastasis diagnosed between April 2018 and January 2019. The study was approved by the Ethics Committee of Tianjin Huanhu Hospital. All patients provided their written informed consent prior to any study procedure.

The inclusion criteria were: (i) diagnosis of NSCLC confirmed by histopathological examination; (ii) first‐ever diagnosis of brain metastasis by MRI and ThinPrep cytologic test; and (iii) no prior treatment for brain metastasis.

### Samples

Samples of CSF and peripheral blood were obtained from all 21 patients. Brain tissues were obtained from five patients after surgical resection. CSF was obtained by lumbar puncture before brain surgery. Blood (10 mL) was collected from an antecubital vein into EDTA tubes. CSF and blood samples were preserved with 10 mL of a patented cell preservation solution (patent #CN201710442744.2) for transport and to ensure high‐quality samples for sequencing. The LiquidBiopsy system focuses on increased capture and molecular analysis of CTCs, including cells undergoing epithelial–mesenchymal transition. CTCs in CSF and blood samples were isolated using this system.

### DNA extraction

Total DNA was extracted from plasma and CSF using the QiAamp Circumstance Nucleic Acid kit (#55114; Qiagen, Hilden, Germany), according to the manufacturer's instructions. Total DNA was extracted from brain lesion specimens using a DNA/RNA Isolation kit (#R6731‐00).

### Next‐generation sequencing (NGS)

The reference library for all three types of samples was constructed using the Ion AmpliSeq Library Kit 2.0 and Ion AmpliSeq Cancer HotSpot Panel v2 (#55114 and #4475346; ThermoFisher Scientific, Waltham, MA, USA) and the Ion Library TaqMan Quantitation kit (#4468802; ThermoFisher Scientific), according to the manufacturer's instructions. PCR amplification was performed using either a Veriti Dx 96‐well Thermal Cycle (#299121427, Thermo Fisher Scientific, Waltham, MA, USA). Samples were prepared using various kits of the Ion system, according to the manufacturer's instructions (Ion One Touch ES [#410122, Thermo Fisher Scientific, Waltham, MA, USA], and Ion One Touch 2 [#2456280‐0935, Thermo Fisher Scientific, Waltham, MA, USA]).

Data were analyzed using Ion Proton software (#DA860001182; Thermo Fisher

Scientific, Waltham, MA, USA). Alignment of NGS reads was performed using TMAP 5.4 software (The ProtPlot Web site). Two types of mutation were screened for

using Torrent VariantCaller 5.4 software: single nucleotide variants (SNVs) and small

insertions or deletions (indels). Variant annotation and filtering were performed using

Ion Reporter 5.2 software, based on the reference genome version GRCh37(hg19), and Oncomine (https://www.oncomine.org/resource/login.html), COSMIC (https://cancer.sanger.ac.uk/cosmic), My Cancer Genome (https://www.mycancergenome.org/), and dbSNP (https://www.ncbi.nlm.nih.gov/snp) databases.

### Statistical analysis

Only descriptive statistics were used. The mutation rate was defined as the number of patients with mutations detected in a specific sample divided by the total number of patients.

## Results

### Characteristics of patients

Among the 21 patients with NSCLC, 10 (47.6%) were male and 11 (52.4%) were female. Mean age was 59.7 ± 9.9 years. A total of 10 (47.6%) patients had leptomeningeal metastases (LM), 11 (52.4%) had brain parenchymal metastases (BPM), 5 (23.8%) patients had *EGFR* mutation‐negative primary tumors at diagnosis, and 13 (61.9%) had positive tumors. A total of 14 (66.7%) patients had previously received treatment with tyrosine kinase inhibitors (TKIs).

### CSF versus blood

**Figure 1:**
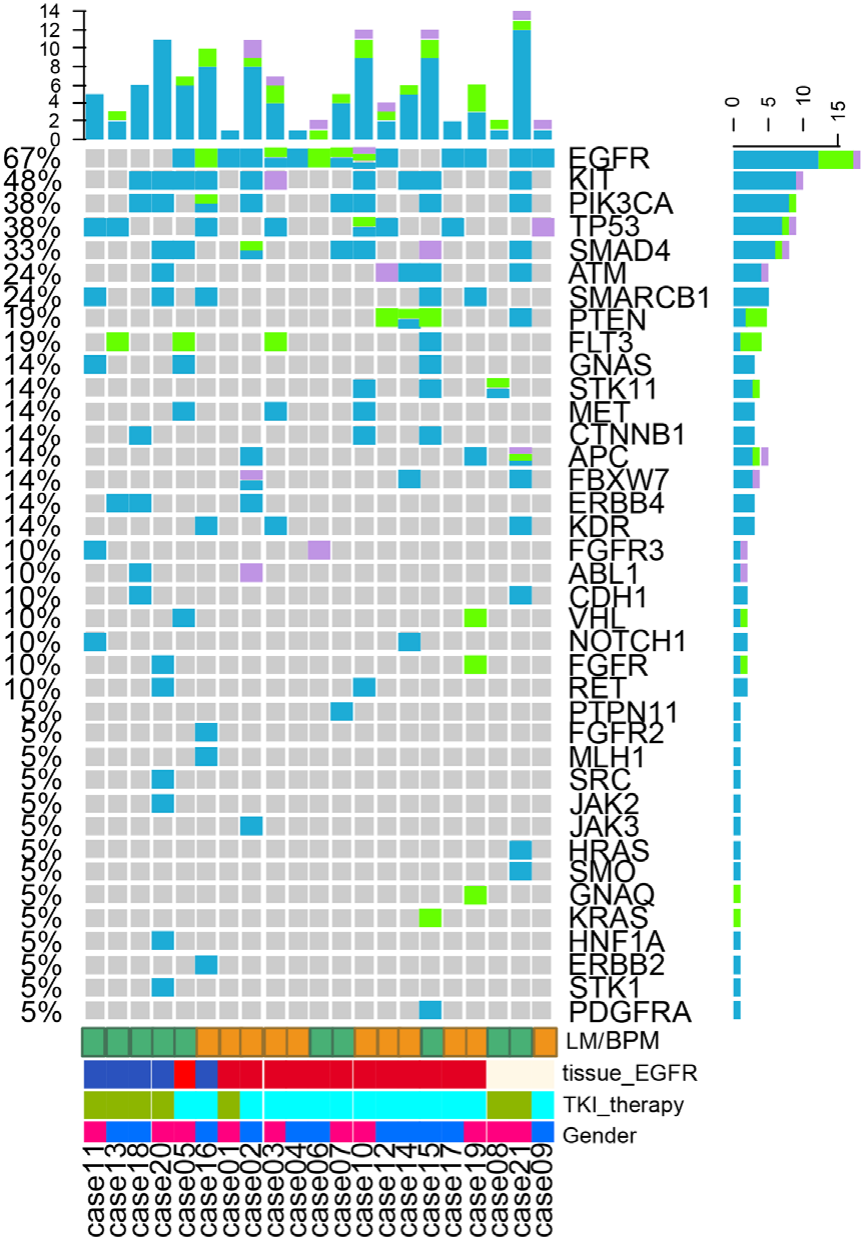
Characteristics and sequencing results of 21 patients with non‐small cell lung cancer and brain metastases based on cerebrospinal fluid (CSF) circulating tumor DNA (ctDNA), plasma ctDNA, and circulating tumor cells (CTCs). BPM, brain parenchymal metastases; LM, leptomeningeal metastases; TKI, tyrosine kinase inhibitor. Sample type (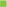) ctDNA, (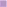) CTC and (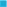) CSF. tissue_EGFR (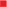) positive, (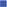) negative and (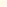) unknown. TKI_therapy (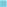) Y and (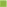) N. Gender (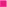) M and (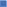) F. (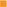) LM and (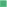) BPM.

Mutations were detected in the CSF ctDNA of 20 patients (95.2%), in the blood ctDNA of 14 patients (66.7%), and in the circulating tumor cells (CTCs) of eight patients (39.0%). The detection rate of *EGFR* mutations in CSF ctDNA was 57.1% (12/21) whereas it was only 23.8% (5/21) in peripheral blood ctDNA and in CTCs (Fig 1). The *EGFR* status in CSF ctDNA was concordant with the *EGFR* status of the primary tumor in 16/18 (88.9%) patients. Mutations were also detected in *KIT*, *PIK3CA*, *TP53*, *SMAD4*, *ATM*, *SMARCB1*, *PTEN*, *FLT3*, *GNAS*, *STK11*, *MET*, *CTNNB1*, *APC*, *FBXW7*, *ERBB4*, and *KDR* (all >10%).

### LM versus BPM

Mutations were detected in the CSF of all 11 patients with LM (100%), as compared with nine of 10 (90%) patients with BPM. *EGFR* mutations were found in the CSF of nine of 11 (81.8%) patients with LM, compared with three of 10 (30%) patients with BPM.

### CSF versus blood and brain lesion tissue

**Figure 2:**
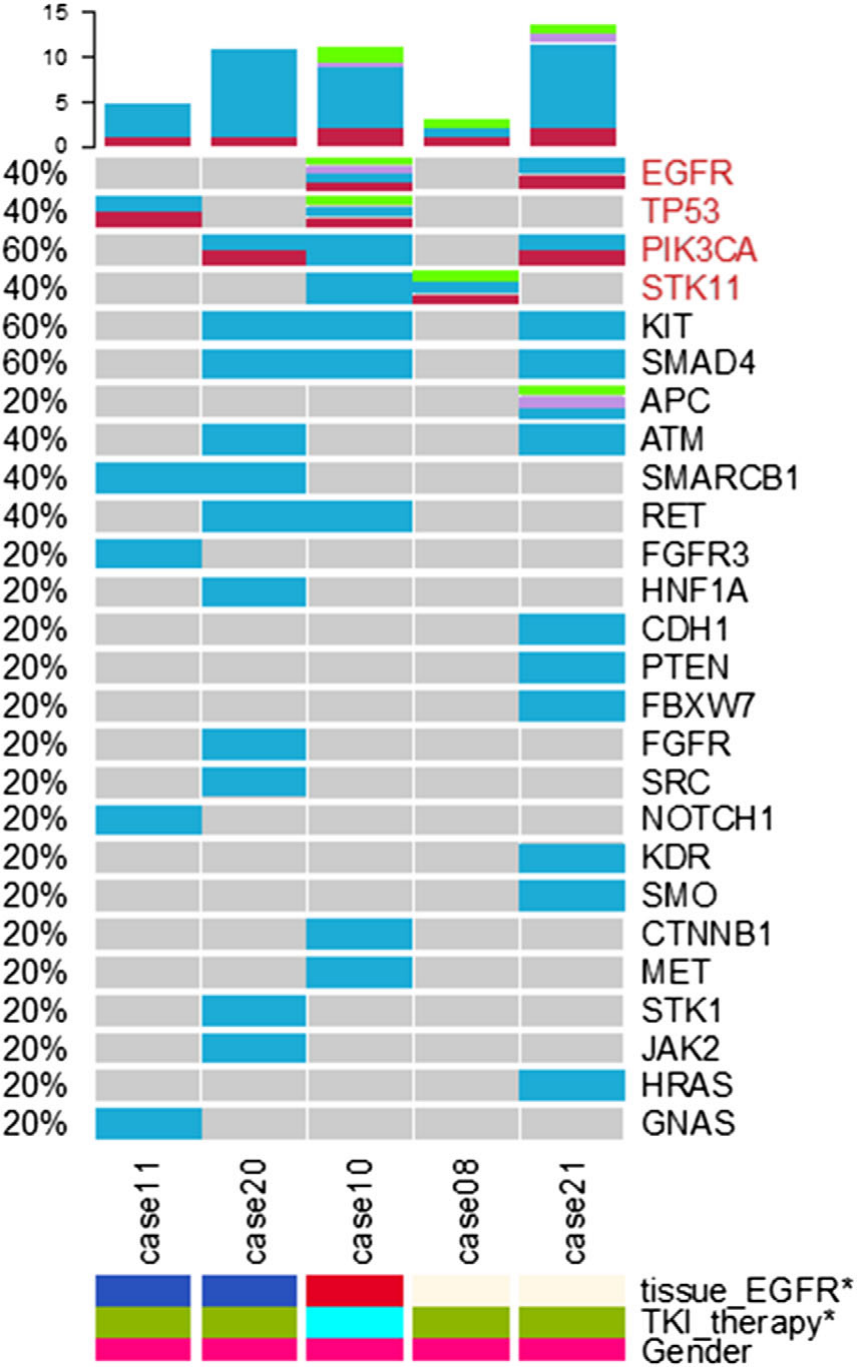
Concordance among cerebrospinal fluid (CSF) circulating tumor DNA (ctDNA), plasma ctDNA, circulating tumor cells (CTCs), and brain lesion. TKI, tyrosine kinase inhibitor. Sample type (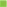) ctDNA, (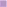) CTC, (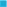) CSF and (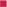) tissue. tissue_EGFR (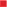) positive, (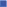) negative and (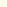) unknown. TKI_therapy (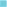) Y and (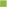) N. Gender (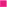) M.

Figure 2 shows the concordance among CSF ctDNA, plasma ctDNA, CTCs, and brain lesions. *EGFR* and *TP53* mutation statuses were consistent in all five patients. There were two patients with lesions and CSF ctDNA *PIK3CA* mutation, but a *PIK3CA* mutation was detected in the CSF ctDNA of one additional patient. One patient had lesions and CSF ctDNA *STK11* mutation, but a *STK11* mutation was detected in the CSF ctDNA of an additional patient. Otherwise, CSF ctDNA mutations were detected in a number of genes, and none were found in brain lesion tissues (Fig 2).

## Discussion

Evaluating the molecular characteristics of brain metastases is limited by the near impossibility of obtaining tissue specimens and by the blood‐brain barrier which prevents ctDNA from entering the blood circulation.^8^ Therefore, in this study, we aimed to compare the results from CSF ctDNA with plasma ctDNA, plasma CTCs, and brain tissue specimens in patients with brain metastasis from NSCLC. The assessment of CSF ctDNA could provide a snapshot of what actually occurs in brain metastases,^11^, ^15^ to more precisely guide therapy.^16^, ^17^, ^18^ Sequencing of CSF ctDNA revealed specific mutation patterns in driver genes among patients with NSCLC and brain metastases. This is the first study comparing CSF ctDNA, blood ctDNA, CTCs, and brain tissue in patients with NSCLC and brain metastases.

Brain parenchymal metastasis may invade the leptomeninges. Variant gene mutations of tumor cells often lead to different proliferation activities, and the location of the resulting metastasis may also differ.^19^ Recently, studies indicated that CSF was more representative of the *EGFR* mutation status in brain metastasis than peripheral blood and could better guide medical treatments.^14^ Nevertheless, that previous study did not evaluate the genetic characteristics of lung cancer with brain metastasis in cotesting of lung tumor tissue, brain metastases, peripheral blood, and CSF. In the present study, we re‐evaluated the clinical application value of CSF in the management of brain metastasis in NSCLC.

The results of gene mutation detection in pulmonary primary lesions, peripheral blood, CSF, and brain tissue are not completely consistent; it is necessary to re‐evaluate the gene detection in brain tumors after brain metastasis has progressed during lung cancer treatment. In the present study, the overall mutation rate was the highest in CSF ctDNA, and the mutations detected were the most complex. Furthermore, mutations were detected in the CSF of 20/21 patients (95.2%), and the detection rate of *EGFR* mutations in the CSF was 57.1% whereas it was only 23.8% in peripheral blood ctDNA and CTCs. The result of *EGFR* mutation in CSF was consistent with that in brain tissue but differed from those in primary lesions at NSCLC diagnosis and peripheral blood. One patient developed metastasis from the brain parenchyma to the leptomeninges, and uncommon mutations were also detected in the CSF.

In terms of *EGFR*‐driven genes, the results from brain lesion tissues were consistent with those from CSF, as supported by Li *et al*.^14^ The detection of *EGFR* mutations in CSF might also reveal uncommon *EGFR* mutations that could be targeted with specific treatments.^20^, ^21^ In addition to *EGFR*, mutations in *KIT, PIK3CA, TP53, SMAD4, ATM, SMARCB1, PTEN, FLT3, GNAS, STK11, MET, CTNNB1, APC, FBXW7, ERBB4, KDR*, and other genes were also frequently detected in CSF and brain tissue. Alteration of the mTOR signaling pathway may be one mechanism in brain metastasis from lung cancer.^22^ These specific mutation patterns might be involved in the migration of cancer cells to the brain; however, additional studies are necessary for confirmation. In addition, differences in *MET* among the primary tumor, peripheral lesions, and brain lesions might guide the treatment as some *MET* mutations lead to resistance to gefitinib but may still respond to crizotinib. The impact of the response in leptomeningeal metastases on NSCLC prognosis outweighs the impact of the responses in peripheral lesions.^13^ Nevertheless, it must be emphasized that multiple sample types should be examined, not only CSF, as the information from each might be complementary. For example, a TKI could be given if an *EGFR* driver mutation is found, irrespective of the location.

It is difficult, often impossible, to obtain brain tissue samples from patients with brain metastasis via nonsurgical methods. Therefore, the brain tissues in this study were all from patients with brain parenchymal metastasis and who underwent surgery. From the standpoint of physiology, when metastasis erodes the leptomeninges, it is easy to create direct contact with the CSF in the ventricle, releasing more ctDNA and even tumor cells. Therefore, it is considered that CSF has importance for guiding the diagnosis and treatment of leptomeningeal metastasis. Indeed, in the present study, the rate of mutation detection was higher in patients with leptomeningeal metastases than in those with brain parenchymal metastases.

The sequencing results of brain tissue were inconsistent with the results of CSF in patients of brain parenchyma metastasis. The reason could be that noise might be present from germline mutations in normal tissue. In addition, for patients with brain parenchymal metastasis, a clinically important tumor mutation in CSF might be associated with high tumor activity, which may warrant close follow‐up. Importantly, the use of a cell preservation solution is crucial to ensure high‐quality liquid biopsy samples for sequencing, as the number of tumor cells and amount of ctDNA are very small and even a small degree of degradation could compromise the results.^23^, ^24^

This study has several limitations. The number of patients was small and only basic descriptive statistics were used. In addition, the impact of each mutation or their combination on the progression of disease and prognosis were not investigated. Brain lesion tissues were not available for all patients and there was no follow‐up to assess the prognosis. The present results should be considered preliminary pending more in‐depth mechanistic studies.

In conclusion, the present study findings suggest that sequencing of CSF ctDNA may reveal specific mutation patterns in driver genes in brain metastases among patients with NSCLC and brain metastasis. CSF is a new form of liquid biopsy that could help in improving the management of patients with brain metastases.

## Disclosure

All authors declare that they have no conflict of interest.
